# Laser Ablated Albumin Functionalized Spherical Gold Nanoparticles Indicated for Stem Cell Tracking

**DOI:** 10.3390/ma16031034

**Published:** 2023-01-24

**Authors:** Dilcele Silva Moreira Dziedzic, Bassam Felipe Mogharbel, Ana Carolina Irioda, Priscila Elias Ferreira Stricker, Thiago Demetrius Woiski, Thiago Neves Machado, Arandi Ginane Bezerra Jr, Katherine Athayde Teixeira de Carvalho

**Affiliations:** 1 Advanced Therapy and Cellular Biotechnology in Regenerative Medicine Department, The Pelé Pequeno Príncipe Research Institute, Child and Adolescent Health Research & Pequeno Príncipe Faculties, Curitiba 80230-901, PR, Brazil; 2 Physics Department, Federal University of Technology, Curitiba 80230-901, PR, Brazil

**Keywords:** gold nanotracers, adipose-derived stromal cells, cytotoxicity

## Abstract

Cell tracking in cell-based therapy applications helps distinguish cell participation among paracrine effect, neovascularization, and matrix deposition. This preliminary study examined the cellular uptake of gold nanoparticles (AuNPs), observing cytotoxicity and uptake of different sizes and AuNPs concentrations in Adipose-derived stromal cells (ASCs). ASCs were incubated for 24 h with Laser ablated Albumin functionalized spherical AuNPs (LA-AuNPs), with average sizes of 2 nm and 53 nm in diameter, in four concentrations, 127 µM, 84 µM, 42 µM, and 23 µM. Cytotoxicity was examined by Live/Dead assay, and erythrocyte hemolysis, and the effect on the cytoskeleton was investigated by immunocytochemistry for β-actin. The LA-AuNPs were internalized by the ASCs in a size and concentration-dependent manner. Clusters were observed as dispersed small ones in the cytosol, and as a sizeable perinuclear cluster, without significant harmful effects on the cells for up to 2 weeks. The Live/Dead and hemolysis percentage results complemented the observations that the larger 53 nm LA-AuNPs in the highest concentrated solution significantly lowered cell viability. The demonstrated safety, cellular uptake, and labelling persistency with LA-AuNPs, synthesized without the combination of chemical solutions, support their use for cell tracking in tissue engineering applications.

## 1. Introduction

Gold nanoparticles (AuNPs) have numerous biomedical applications [1]; among diagnostics [2], cancer therapeutic use [2,3,4,5,6,7], enhancement in radiological imaging [8], improvement of transplantable matrices properties [9], as biotracers [3,10,11,12,13], for drug delivery [3,14], etc. Even though Nano Particles (NPs) can be prepared from metal and non-metal polymers, bioceramics, etc., the detection methods for metallic NPs could facilitate their observation with non-invasive detection methods, using tracing agents with high resolution and longer detection time, without intensity attenuation [15]. Nano-drug delivery in cancer therapeutics with NPs is greatly indicated because of the observed accumulation of NPs in tumor tissues through a passive retention mechanism, referred as Enhanced Permeability and Retention effect, by defective tumor vasculature and decreased lymphatic drainage of tumors [4]. At the cellular level, the recognition of the albumin on the surface of NPs by cell receptors facilitates cellular uptake and increases their bioavailability, through specific receptors of tumor cells [16,17], and leads to higher NPs distribution in the tumor region by facilitating transcytosis across the endothelial barrier via the albumin receptor [17,18].

Gold nanoparticles can be synthesized through various methods, biological, physical, and chemical synthesis techniques [19,20]. Many studies with AuNPs have used particles prepared through chemical synthesis [10,13,15,21,22,23,24,25,26,27,28,29,30,31]. However, authors have suggested the necessity of reducing some chemical components to guarantee stability to the particles, lower cell viability impairment [28], and lower cytotoxicity [26,32].

All components involved in AuNPs processing and their characteristics, such as composition, shape, size, concentration, coating, functionalization, etc., have important consequences on in vitro and in vivo responses. Different AuNPs shapes and sizes can be produced through chemical, thermal, electrochemical, or photochemical synthesis; as nanospheres, nanorods [15], nanostars [10,21], and anisotropic gold nanoparticles [33]. The preparation of more biocompatible gold nanoparticles from natural sources have also been explored, such as the physical method of Pulsed Laser Ablation in Liquids (PLAL) [34,35,36,37,38], which does not require the components used in chemical synthesis, avoiding any residual toxic contaminants that could affect biological responses.

Gold nanoparticles are considered safe because their cores are inert, and their surface presents easy functionalization by binding to biomolecules indicated to improve biocompatibility. Nanoparticles form a complex molecular shield with proteins on the surface, referred as “protein corona”, when entering biological systems, in contact with blood and body fluids. Albumin is the most abundant blood protein, with relevant coating properties in drug delivery and regenerative medicine [17,39], enhancing the stability and biocompatibility of drug carriers and nanoparticles [17,18], and the albumin “capping” of gold nanoparticles has improved compatibility, bioavailability, circulation times, lowered toxicity, and selective bioaccumulation [16].The serum albumin corona formation onto gold nanoparticles, its conformational characteristics, molecular dynamics changes and its properties have been investigated by molecular dynamic simulation and atomistic force field [40], and the albumin corona presence was investigated with electron paramagnetic resonance and Raman spectroscopies [41]. The thermodynamic nature and the mechanisms of the binding process interaction between noble metal NPs with serum albumins depend partly on the size, shape, and metal type, while the chemical structure and the charge of the stabilizing ligands affect the change in optical features [42]. The proteomics of the protein corona characterize the NPs’ processing, bio-distribution, toxicity, and clearance, and can also be used as a probe for diseases [43]. Authors have also observed that the albumin coating on the surface of nano-carriers may reduce the host’s rejection by modulating clearance rate, detection by macrophages, and promote immune system evasion by attenuating acute immune and inflammatory responses [39].

The protein coating adsorption from a biological fluid or serum-containing medium stabilizes the nanoparticles [41] and decreases their agglomeration in cell culture, affecting biocompatibility/toxicity, intracellular uptake and blood circulation [34,44,45]. Surface modifications of functionalized AuNPs present an impact on their cellular uptake, as cell-penetrating peptides increase NPs internalization [15,46], and their biomedical application [14,15]. Bovine Serum Albumin (BSA) has been used to enhance the biocompatibility of AuNPs, due to high binding affinity to gold nanoparticles, and broad pH stability [26,34].

However, physicochemical characteristics, such as size and charge, of gold nanoparticles prepared by laser ablation modify after incubation in serum-containing culture medium [34]. The mechanisms for AuNPs cell interaction and internalization have been hypothesized, initiated by outer membrane contact/adsorption promoting different signaling cascades, structural changes at the cell surface, and subsequent internalization [46,47].

To understand the contribution of stem cells to tissue repair, cell tracking is important to distinguish among paracrine effects, neovascularization, and matrix deposition. Cell labeling with gold nanotracers has great potential for non-invasive in vivo detection methods, such as Micro-Computed Tomography [11,48,49], and ultrasound-guided photoacoustic imaging [10,13,50,51], which could be employed for tracking the transplanted cells over long periods with an optically absorbing contrast agent because of their cytocompatibility and strong optical absorption in the near-infrared region [23].

This preliminary in vitro study elucidated the cellular response, for the selection of laser-synthesized spherical AuNPs indicated for longitudinal cell tracking, aimed at monitoring cell-based therapies with Adipose-derived stromal cells (ASCs). The AuNPs used in the present study were prepared with the Nd:YAG (Neodymium laser emission-doped yttrium aluminum garnet) laser ablation of a high-purity gold pellet target, without the addition of chemical elements. The ASCs were selected as possible cell sources for enhancing in vivo bone regeneration, as previously observed when associated to a collagenous matrix carrier [52,53], and for future cell tracking by non-invasive methods.

## 2. Materials and Methods

### 2.1. Preparation and Characterization of Gold Nanoparticles

**Figure 1:**
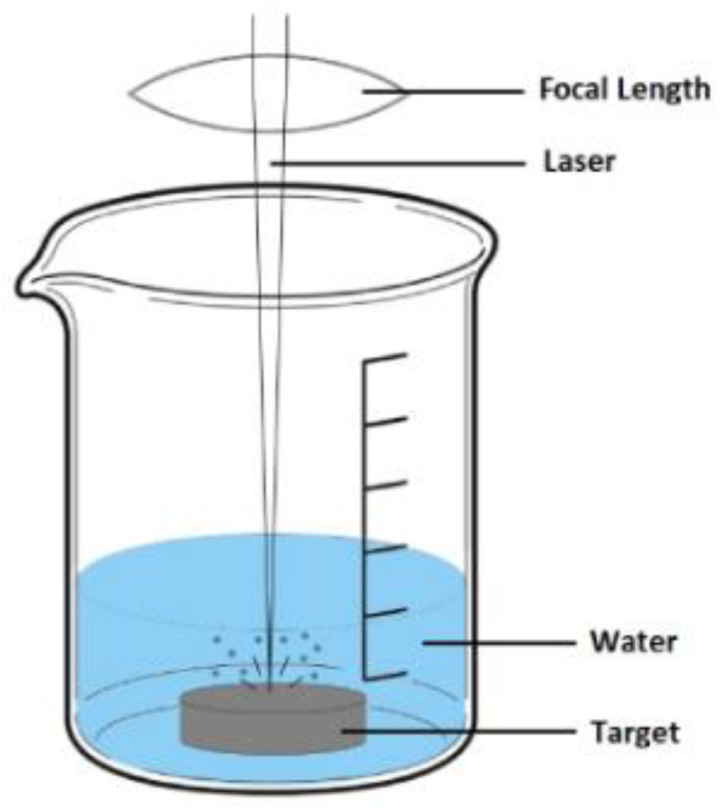
Experimental setup used for nanoparticles’ synthesis by Pulsed Laser Ablation in liquid technique: laser pulse irradiation onto gold target immersed in water causes the gold outer surface atoms to vaporize and condense in the water, forming the nanoparticles.

AuNPs were synthesized by Pulsed Laser Ablation in Liquids (PLAL), as thoroughly explained previously [38], at the Federal University of Technology (UTFPR, Curitiba, PR, Brazil). High-purity gold (>99.99%) pellet targets immersed in 10 mL of double distilled water were irradiated with an Nd: YAG laser (Quantronix, Model 117, Cascade Laser Corporation, Newberg, OR USA) operating at 1064 nm, Q-switched at 1.6 kHz, and delivering 200 ns pulses. The laser was focused onto the gold target at 1 mm below the water surface with a 5 cm focal lens to a spot size of 40 μm (Figure 1). The target was randomly moved for 5 min in the focusing plane to attain identical surface conditions during laser ablation. Laser energy was adjusted at 3 mJ and at 1.5 mJ so two independent batches (AuNPs-1 and AuNPs-2, respectively) with different nanoparticle size distributions were synthesized. Under these experimental conditions, Au-target ablation rate was measured at 1.0 mg/min and 0.6 mg/min, respectively.

Two colloidal solutions of gold nanoparticles were prepared, with an average size of 2 nm (0.18 mg of Au) and 53 nm (0.05 mg of Au). The solution with smaller nanoparticles was diluted so that both had the same molarity. Bovine Serum Albumin (BSA A7906, Sigma, St. Louis, MO, USA) was added to the solutions of each size (final concentration 0.05%). The stabilization of gold nanoparticles in the presence of BSA [41] is indicated particularly for those of larger sizes, which tend to be less stable and aggregate over time.

The AuNPs colloidal solutions were characterized by UV/Vis spectroscopy (Ocean Optics, Model USB2000+, StellarNet Inc., Tampa, Florida, USA; 200 nm to 1000 nm), Dynamic Light Scattering (DLS) (Microtrac, Nanotrac Ultra model, Microtrac Retsch GmbH, Haan, North Rhine–Westphalia, Germany), Transmission Electron Microscopy (TEM) (JEM-1400 Flash Electron Microscope, JEOL, Akishima, Tokyo, Japan) and Zeta Potential (Zetasizer Malvern, Malvern Panalytical, Malvern, Worcestershire, UK).

After the AuNPs dilution, functionalization, and characterization, the now referred to as Laser ablated Albumin functionalized AuNPs solutions (LA-AuNPs) were kept at room temperature and protected from light. Before application in cell culture there was no detectable sign of precipitation or color change, the solutions were filtered in a 0.22 μm mesh and diluted in the culture medium.

### 2.2. Adipose-Derived Stromal Cell Collection and Expansion

The methodology used in this study to collect and isolate Adipose-derived stromal cells (ASCs) was adapted from a previous study [54] and followed an experimentation protocol approved by the Ethical Committee in the Use of Animals (CEUA) of the Pequeno Príncipe Complex (Curitiba, Paraná, Brazil). Adipose tissue samples were collected from the biopsy of rats (320 g), with the removal of inguinal fat pads, after intraperitoneal anesthesia with 50 mg·kg^−1^ ketamine and 6.6 mg·kg^−1^ xylazine, followed by their euthanasia with intracardiac administration of Thiopental 75 mg·kg^−1^. Adipose tissue samples, handled in a Class II laminar flow cabinet, were washed twice with sterile phosphate buffered saline (PBS) at 37 °C containing 2% penicillin/streptomycin (P/S). The tissue was macerated onto a sterile petri dish and digested with collagenase type I 0.075% for 30 min under agitation. Enzymatic activity was inactivated by equal volume addition of standard culture medium Dulbecco’s Modified Eagle’s Medium (DMEM), with 10% Fetal Bovine Serum (FBS) and 1% P/S. The adipose tissue samples were first centrifuged at 1200 rpm for 10 min, the supernatant was discarded, resuspended in PBS, and centrifuged again to isolate the stromal vascular fraction (SVF). The SVF was resuspended, filtered through a 100 μm sieve, centrifuged again, and resuspended in standard culture medium. Cells from the SVF were incubated at 1 × 10^5^ cells.cm^−2^ in standard medium for cell proliferation at 37 °C and 5% CO_2_. The culture medium was changed every 72 h, when non-adherent cells were removed, until observation of cell proliferation with approximately 80% confluence on the flask surface. Cells were transferred after trypsinization to other flasks at 1 × 10^3^ cells.cm^−2^ for proliferation and at the third passage to tissue culture plates for nanoparticle experiments. After 80% of cell confluence on the tissue culture plates indicated for each experiment, the defined dilutions of nanoparticles in culture medium were placed on the wells and incubated.

### 2.3. Cell Incubation with Nanoparticles

Dilutions of LA-AuNPs with an average size of 2 nm and 53 nm in diameter in cell culture medium were prepared with 0.05 mg·mL^−1^ of gold; 1:1 (127 µM), 1:2 (84 µM), 1:5 (42 µM), and 1:10 (23 µM), and agitated by hand. The total volume used was 150 µL in each well of 96-well plates, and 1 mL in each well of 24-well plates. The ASCs were incubated at 37 °C and 5% CO_2_ with the different LA-AuNPs dilutions for 24 h and compared to untreated cells as control, in quadruplicate samples and double or triplicate testing. After 24 h the LA-AuNPs-containing medium was removed, the wells were washed with PBS 3 times, and new culture medium was added for cultivation during the recovery period, depending on the experiment. Medium was changed every 3 days, and the preservation of ASCs labeled with gold nanotracers was investigated during the cell detachment procedure after 2 weeks.

### 2.4. Continuous Monitoring during LA-AuNPs Incubation

The effect of incubating the cells with gold nanoparticles was investigated by continuous observation for 24 h on 24-well plates in In Cell Analyzer 2000 (GE Healthcare Europe GmbH, Freiburg in Breisgau, Baden-Württemberg, Germany). Cells were incubated without LA-AuNPs for control, and with LA-AuNPs in dilutions of 1:1, 1:2, 1:5, and 1:10, and 1 mL was deposited in each well. Cell culture of three cell samples in quadruplicate was used to monitor overall cell area, morphology, motility, and uptake in continuous time for 24 h, through 145 serial photographs with 10× objective.

### 2.5. Cytotoxicity Assay

The cytotoxicity of LA-AuNPs was evaluated using the Live/Dead test in 96-well plates, after incubation of cells with LA-AuNPs, and control without LA-AuNPs. Two compounds for fluorescence detection were added to the culture, which interact with either live or dead cells, along with Hoechst nuclear marker (H33342, Bristol, UK). Viable cells were labeled with Acetomethoxyl calcein (calcein-AM), whose cleavage by intracellular esterase promotes the release of calcein, with green fluorescence (fluorescence wavelength detected by FITC channel). The addition of ethidium-1 homodimer (EthD-1) promotes the labeling of dead cells, in which the loss of membrane integrity allows binding of nucleic acids with Eth-1, and detection of red fluorescence (fluorescence wavelength detected by Texas Red channel). Live/Dead test solution was prepared with PBS and addition of the markers provided in the Molecular Probes Invitrogen kit (Thermo Fisher Scientific, Eugene, OR, USA; 0.0003 mM calcein-AM, 0.0006 mM EthD-1, Hoechst 0.002 mg/mL). Wells were washed twice with PBS after culture medium was removed, solution was placed and the plate incubated for 45 min, before observation of fluorescence emission in In Cell Analyzer 2000. The data from the Live/Dead assays were analyzed using Shapiro Wilk, Kruskal–Wallis, and multiple comparisons tests (STATISTICA software Version 10, StatSoft, Dell). Differences were considered significant when *p* <0.05.

### 2.6. Cell Observations

General cell morphology was visualized after fixation with 4% paraformaldehyde through staining with 4% Giemsa solution for 1 min, washing with distilled water and natural drying. For cytoskeleton β-actin immunocytochemistry, after fixation with 4% paraformaldehyde, cells were permeabilized with 100% methanol at −20 °C for 15 min, and 0.5% Triton X-100 (Amresco Inc., Solon, OH, USA) in PBS for 15 min. Non-specific binding was blocked with 1% goat serum (Sigma-Aldrich, St. Louis, MO, USA) for 30 min. After each incubation step, the samples were washed thrice under gentle agitation on a horizontal shaker table for 5 min. The primary antibodies anti β-actin (mouse monoclonal anti βACT antibody, working dilution of 1:1000, MA1-140, Thermo Fisher Scientific, Rockford, IL, USA) were incubated overnight under refrigeration and were followed by corresponding goat anti-mouse (IgG FITC, working dilution of 1:1000, Sigma Aldrich, St. Louis, MO, USA) for 1 h at room temperature. Hoechst’s solution (H3569, 2μL/mL for 5 min, Invitrogen, Eugene, OR, USA) was used for nuclear staining. Cultures were examined under a phase contrast microscope (Zeiss Axio Vert.A1, Zeiss, Jena, Thuringia Land, Germany), equipped with an AxioCam MRC camera (Zeiss, Jena, Thuringia Land, Germany).

### 2.7. Erythrocyte Hemolysis Assay

The erythrocyte hemolysis test [55] was used to analyze the cytotoxicity of LA-AuNPs after a 3 mL sample of human donor blood was obtained by venipuncture and collected in a tube containing 3.2% sodium citrate. Centrifuging the tube at 2000× *g* for 5 min allowed the separation of erythrocytes in the lower layer and discarding, by gentle pipetting, the plasma in the upper layer. The erythrocyte layer was washed four consecutive times, by resuspension in saline solution, with four times the volume of erythrocytes. Gentle manual inversion of the tubes allowed the mixing of serum and erythrocytes before each centrifugation (2000× *g* for 5 min), followed by discarding the supernatant. The final 10% erythrocyte suspension was prepared with saline. Triplicates of the LA-AuNPs dilutions were prepared with saline solution (380 µL each) in a microtube for centrifugation (0.5 mL). Negative control was prepared with saline solution and positive control with distilled water, without LA-AuNPs. Erythrocyte suspension (20 µL) was added to each bottle, incubated for 1 h at 37˚C with gentle shaking, and the samples were centrifuged for 5 min, 2000× *g*. The absorbance of 200 µL of the supernatant, placed in wells of a 96-well plate, was measured in an ELISA reader at 405 nm to determine the percentage of hemolysis, considering 100% of hemolysis caused by distilled water. Percentage of hemolysis was calculated using the equation: (mean optical density of the sample—mean optical density of the negative control)/(mean optical density of the positive control—mean of the optical density of the negative control). The data from the hemolysis test were analyzed using Shapiro Wilk, ANOVA and Tukey tests (STATISTICA software Version 10, StatSoft, Dell). Differences were considered significant when *p* <0.05.

## 3. Results

**Figure 2:**
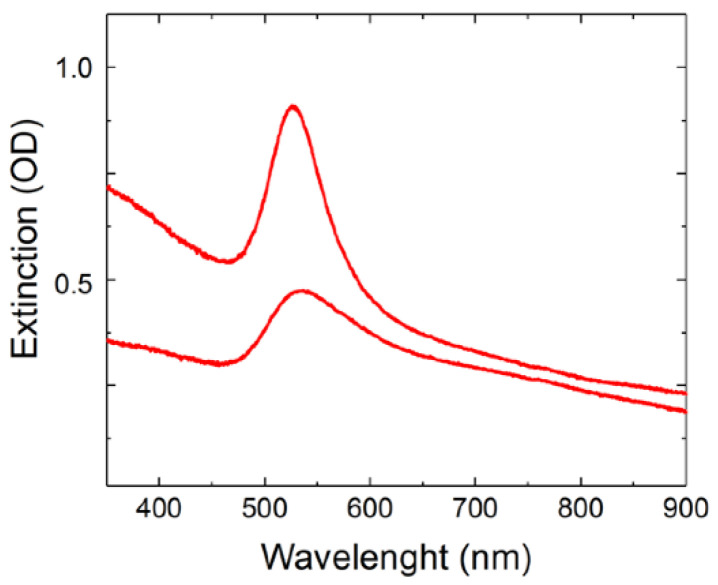
UV–Vis characterization of AuNPs synthesized by Pulsed Laser Ablation in Liquids (PLAL). Two independent batches were analyzed: AuNP-1 (top) with plasmon peak at 523 nm, and AuNP-2 (bottom), with plasmon peak centered at 535 nm, optical density (OD). The extinction spectra are compatible with spherical-shaped AuNPs.

**Figure 3:**
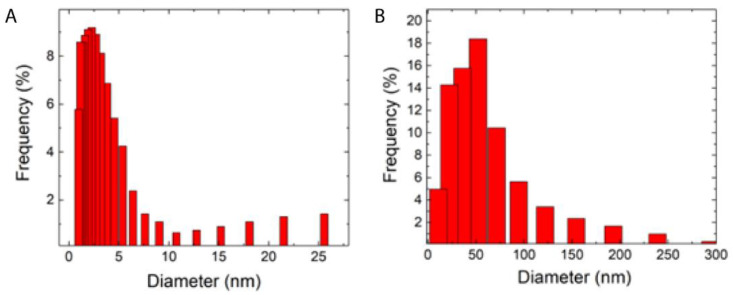
Dynamic Light Scattering (DLS) data for the characterization of AuNPs synthesized by Pulsed Laser Ablation in Liquids (PLAL) presented in number mode. The average diameters were 2 nm for AuNP-1 (**A**) and 53 nm for AuNP-2 (**B**).

**Figure 4:**
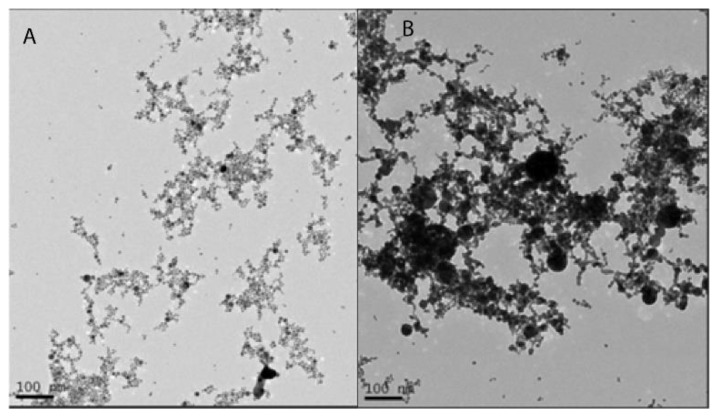
Transmission Electron Microscopy (TEM) images of AuNP-1 (**A**) and AuNP-2 (**B**) demonstrated spherical morphologies and diameter variation, allowing the observation of differences in size and size distribution of both batches, which agrees with PDI and DLS results. Scale bars 100 nm.

UV/Vis spectroscopy was performed to determine the presence of Au in the colloidal solutions. The size and morphology of the AuNP were assessed by DLS and TEM. The zeta potential was used to evaluate colloidal stability. The two different batches of AuNP synthesized by PLAL showed a characteristic spherical-shaped gold nanoparticle plasmon peak around the green region of the spectrum [37] (Figure 2). According to the UV–Vis spectroscopy analysis, AuNP-1 exhibited a plasmon peak centered at 523 nm, whereas AuNP-2 presented a plasmon peak centered at 535 nm. The red shift indicated that AuNP-2 presented larger average diameters as compared to AuNP-1, which agreed with the DLS results (Figure 3). AuNP-1 presented a size distribution ranging from 1 nm to 26 nm (5 nm standard deviation), with a higher prevalence of diameters from 1 nm to 5 nm, and an average size of 2 nm (Figure 3A). On the other hand, AuNP-2 presented a broader size distribution from 30 nm to 300 nm (31 nm standard deviation), with an average size centered at 53 nm (Figure 3B). The breadth or dispersion of size distribution is given by the polydispersity index (PDI), which is defined by the square of the standard deviation divided by the square of the mean. Therefore, PDI for AuNP-1 equals 0.16, whereas AuNP-2 presents a larger PDI of 0.34. The TEM images indicated that the AuNP were spherical and heterogeneous in size, with AuNP-1 presenting smaller nanoparticles as compared to AuNP-2 (Figure 4), which is in accordance with the DLS results, showed in Figure 3. DLS data are presented in number mode, following the DLS equipment (Microtrac, Nanotrac Ultra model) instructions, and software available to the equipment. The solvent used was water, as described in the “Materials and Methods” section. The number mode carries data significance given that the nanoparticles are spherical, the colloidal optical properties (index of refraction) are known, and correlation functions are repeatable. To complete AuNPs characterization, the zeta potential values were −31.91 ± 1.37 for AuNP-1, and −29.78 ± 1.65 for AuNP-2, indicating high stability of both batches of colloidal solutions.

**Figure 5:**
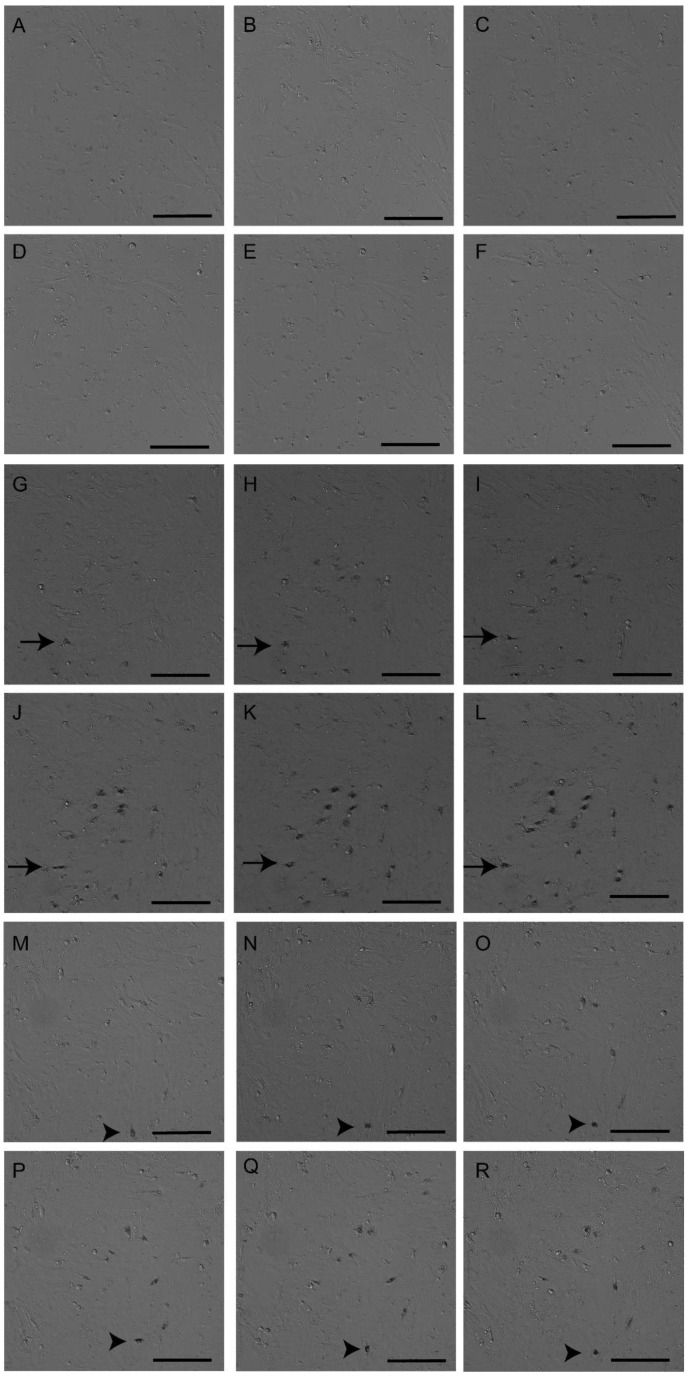
Incubation of Adipose-derived stromal cells (ASCs) with Laser ablated Albumin functionalized AuNPs (LA-AuNPs) for 24 h. Serial images of control without LA-AuNPs (**A**–**F**), cells exposed to 2 nm LA-AuNPs in the dilution 1:1 (**G**–**L**), and 53 nm LA-AuNPs in the dilution 1:1 (**M**–**R**). The sequence of images from after 6 h and 40 min (**A**,**G**,**M**), 10 h (**B**,**H**,**N**), 13 h and 20 min (**C**,**I**,**O**), 16 h and 20 min (**D**,**J**,**P**), 20 h (**E**,**K**,**Q**), and 23 h and 20 min (**F**,**L**,**R**), demonstrated a gradual increase in LA-AuNPs cell labelling and their motility during the incubation (arrows indicate one cell labelling with 2 nm LA-AuNPs, and arrowheads indicate one cell labelling with 53 nm LA-AuNPs). Objective 10×, scale bars 200 µm.

Cell culture with the addition of LA-AuNPs (average sizes 2 nm and 53 nm) and incubation for 24 h allowed the observation of cell culture and LA-AuNPs uptake in continuous time (IN Cell Analyzer), when endocytosis of nanoparticles of two sizes was recorded. General cellular morphological changes during LA-AuNPs exposure were not different from random cell movement of control cell culture, without nanoparticles. Continuous serial images verified cell shape alteration during cell motility, modifying through various elongated fibroblastic shape, and to more circular shape with less surface adhesion. The culture medium change to that with dispersed gold nanoparticles was gradually followed by nanoparticle adsorption onto the cells attached to the bottom of the well. The time that elapsed between LA-AuNPs incubation and cellular uptake was dependent on particle adsorption. Gradual cellular uptake was better visualized in serial photographs of greater LA-AuNPs concentrations for both sizes (Figure 5). The LA-AuNPs uptake was observed as particles were deposited onto the upper cell membrane, adsorbed to the membrane, and internalized, forming a large cytosol cluster, as if the particles were wrapped together, or as a dispersion of small clusters in the cytoplasm.

**Figure 6:**
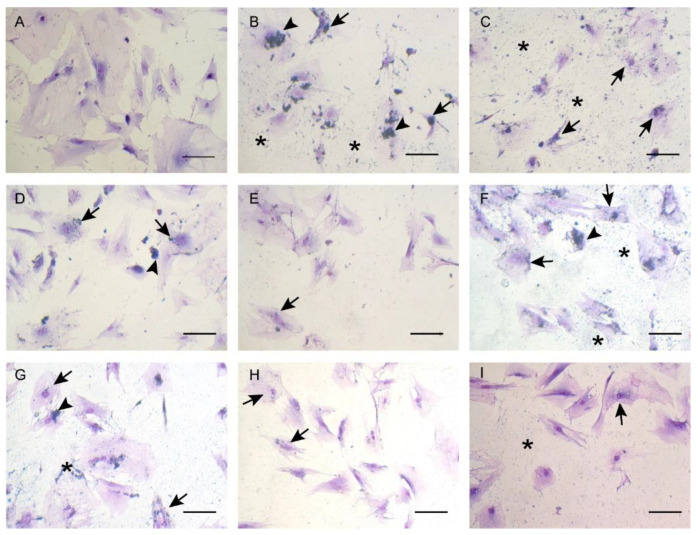
Incubation of Adipose-derived stromal cells (ASCs) with Laser ablated Albumin functionalized AuNPs (LA-AuNPs) for 24 h resulted in fine dispersion of particles on well surface (asterisk), LA-AuNPs intracellular uptake (arrow), and clusters on cell surface (arrowhead). Control without LA-AuNPs (**A**), cells exposed to 2 nm LA-AuNPs (**B**–**E**), and 53 nm LA-AuNPs (**F**–**I**), in the dilutions 1:1 (**B**,**F**), 1:2 (**C**,**G**), 1:5 (**D**,**H**), and 1:10 (**E**,**I**). Giemsa staining, objective 20×, scale bars 100 µm.

LA-AuNPs were observed in the cytosol as a large cluster adjacent to the nuclear membrane, dispersed in the cytosol as small clusters, as clusters outside the cellular membrane, and dispersed on the intercellular space of the well surface. The LA-AuNPs were discerned intracellularly and possibly associated with the cell membrane (Figure 6).

After incubation with 2 nm and 53 nm LA-AuNPs for 24 h, the metabolic activity of cells was compared to untreated cells by optical density percentage in MTT assays (Supplementary materials). The reduction of the yellowish tetrazolium salt (MTT, [3-(4,5-dimethylthiazol-2yl)-2,5-diphenyl tetrazolium]), into formazan blue crystals (E, Z-1-(4,5-dimethylthiazol-2-yl)-1,3-diphenylformazan) insoluble in aqueous solution, indicates the mitochondrial cell function (dehydrogenases). The contradictory results of the MTT method used are presented in the Supplementary materials (Figure S1), and the possible interference of particles on the well surface, adhered to cell surface or due to the cellular uptake cannot be ruled out to be directly related to the increase in optical density in MTT, but not to the increase in cell viability.

**Figure 7:**
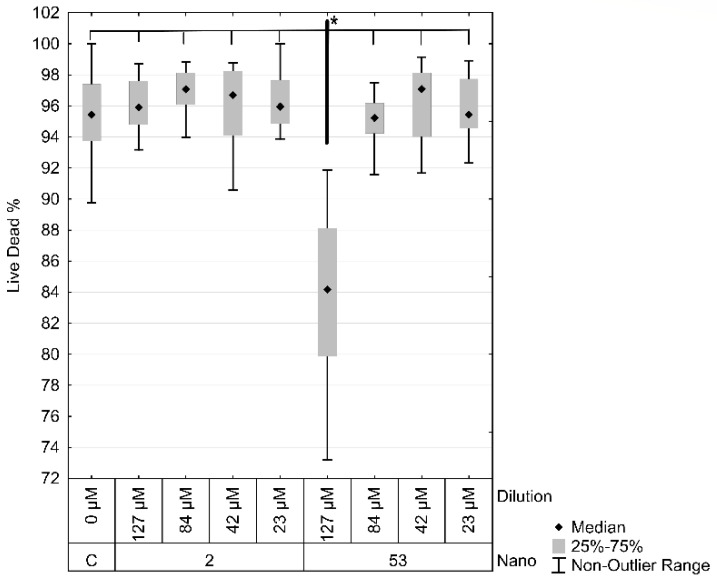
Effect of Laser ablated Albumin functionalized AuNPs (LA-AuNPs) on Adipose-derived stromal cells (ASCs) on cellular cytotoxicity assessed by Live/Dead assay, presented as survival percentage. Cells were exposed for 24 hours to four different concentrations of nanoparticles with two different particle sizes, 2 nm, and 53 nm, in four dilutions: Control (C), 1:1 (127 µM), 1:2 (84 µM), 1:5 (42 µM) and 1:10 (23 µM). Data analyzed by Kruskal-Wallis’s test and represented as median ± 25%–75%. * Significantly lower viability.

Results of the Live/Dead assays were analyzed after obtaining the percentage and number of cells, alive, dead and with apoptosis, in wells and fields. Kruskal–Wallis’s test of Live/Dead assays demonstrated a significant difference and multiple comparisons observed differences between 53 nm 1:1 and all the other groups, including control, all dilutions of 2 nm, and the other three 53 nm dilutions (Figure 7).

**Figure 8:**
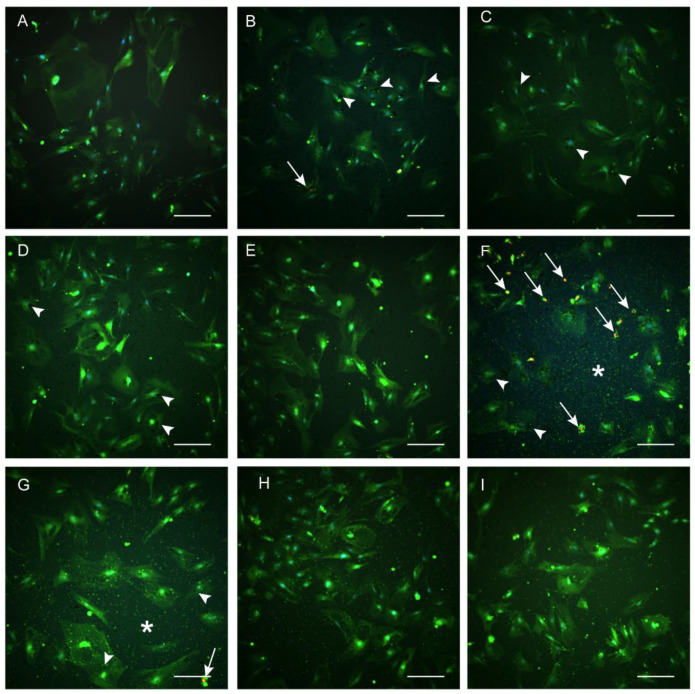
Images from the Live/Dead assays. Adipose-derived stromal cells not incubated with Laser ablated Albumin functionalized AuNPs (LA-AuNPs) ((**A**), control), exposed for 24 h to 2 nm LA-AuNPs (**B**–**E**), and 53 nm LA-AuNPs (**F**–**I**), in the dilutions 1:1 (**B**,**F**), 1:2 (**C**,**G**), 1:5 (**D**,**H**), and 1:10 (**E**,**I**). Fluorescent labeling of viable cells with calcein (green), dead cells marked with Eth-1 (red, arrows in (**B**,**F**)), and nuclei marked with Hoechst (blue). Nanoparticles detected as fine dispersion on well surface (asterisk), and cellular uptake observed as black clusters in the cytoplasm (arrowhead). Objective 20×, scale bars 200 µm.

Images from Live/Dead assays allowed the observation of LA-AuNPs cellular uptake, green cells were considered live, and red cells were considered dead (Figure 8). Incubation of ASCs with LA-AuNPs for 24 h resulted in uptake of dispersed small clusters or a single large cluster (Figure 8). Intercellular spaces with LA-AuNPs dispersion were observed on the well bottom (Figure 8).

**Figure 9:**
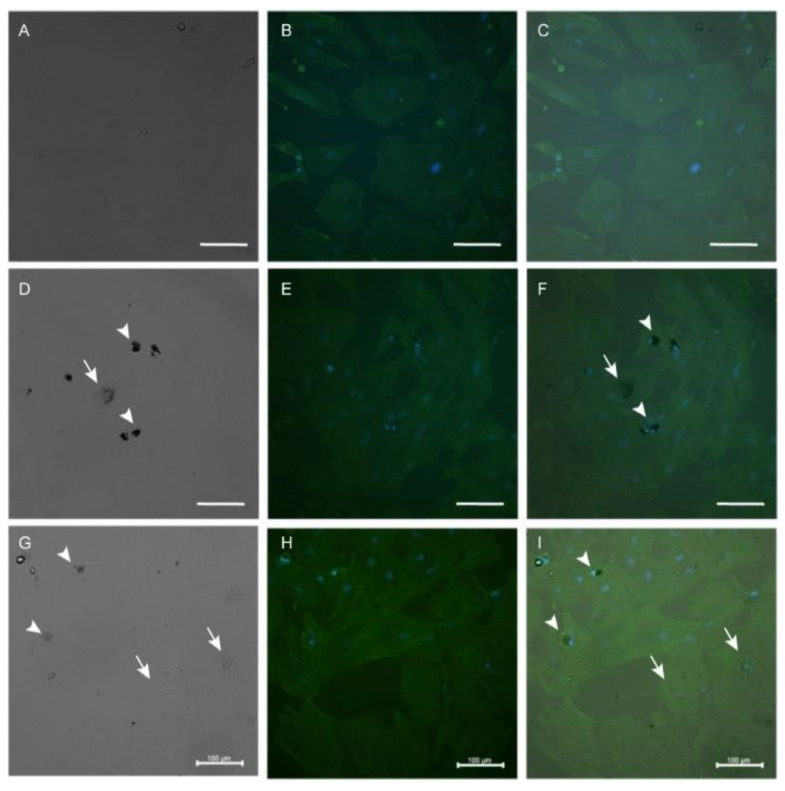
Immunolabeling of β-actin of Adipose-derived stromal cells with 1 week of recovery after 24-h incubation with LA-AuNPs, with uptake and retention of nanoparticles: control (**A**–**C**), 2 nm LA-AuNPs 1:1 (**D**–**F**), 53 nm LA-AuNPs 1:1 (**G**–**I**), on corresponding bright field (**A**,**D**,**G**), fluorescence (**B**,**E**,**H**), and merged (**C**,**F**,**I**) images. Uptake of LA-AuNPs of both sizes were observed as a black cluster in the area adjacent to the nucleus (arrowhead), or more evenly distributed as small clusters in the cytoplasm (arrows). Immunolabeling of β-actin with FITC (green), and nuclei marked with Hoechst (blue). Objective 40×, scale bars 100 µm.

**Figure 10:**
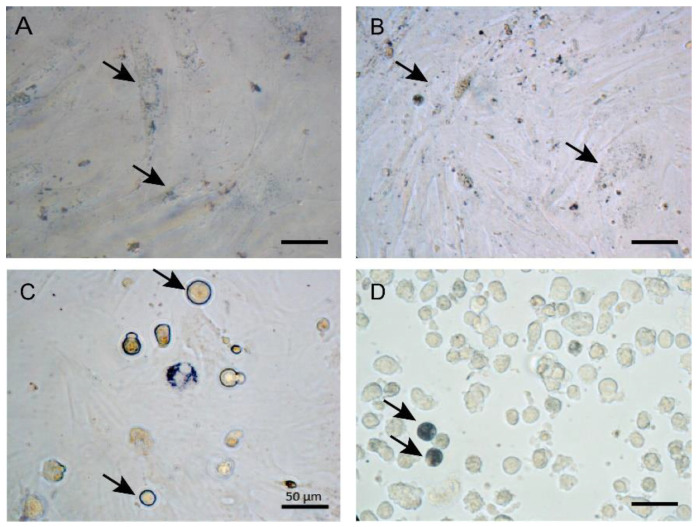
Adipose-derived stromal cells uptake and retention of Laser ablated Albumin functionalized AuNPs (LA-AuNPs), 2 weeks of recovery after incubation with LA-AuNPs for 24 h. ASCs nanotracer preservation with 2 nm LA-AuNPs 1:1 (**A**), 53 nm LA-AuNPs 1:1 (**B**). Enzymatic digestion of 2 nm LA-AuNPs 1:1 during the trypsin digestion (**C**), and after trypsin digestion (**D**). Nanoparticles uptake (arrows) observed dispersed on cells cytoplasm (**A**,**B**), as a halo on the cytoplasm of instantly detached cells (arrows, (**C**)), and clusters on advanced detached cells (arrows, (**D**)). Objective 40×, scale bars 50µm.

After ASCs incubation with the highest LA-AuNPs concentration for 24 h and 1 week of recovery the cells remained labeled with LA-AuNPs as nanotracers, their morphology was similar to control, and actin filament disruption was not detected (Figure 9). Cell culture allowed the preservation of nanoparticle labeling for up to 2 weeks of culture recovery, before and during cell culture trypsinization for cell detachment from the substrate (Figure 10).

**Figure 11:**
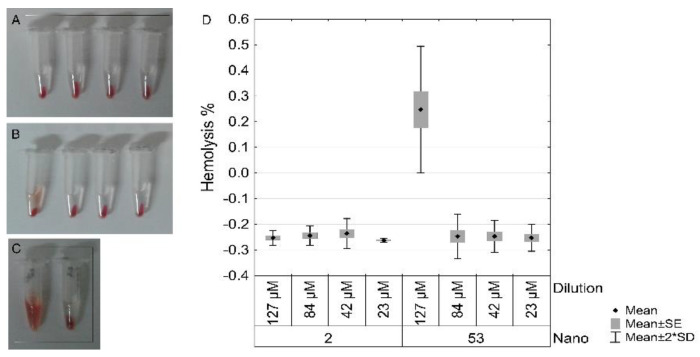
Samples from the groups used in the hemolysis test, after Laser ablated Albumin functionalized AuNPs (LA-AuNPs) incubation with erythrocytes, centrifugation, and removal of supernatant for optical density analysis. Vials with erythrocytes exposed to nanoparticles 2 nm LA-AuNPs (**A**), 53 nm LA-AuNPs (**B**), in the dilutions 1:1 (127 µM), 1:2 (84 µM), 1:5 (42 µM) and 1:10 (23 µM) from left to right, respectively. Positive control was prepared with distilled water and negative control with saline solution (**C**). Data from the hemolytic activity percentage were analyzed by ANOVA (**D**), represented as mean ± SE (standard error) ± 2 SD (standard deviation).

In the erythrocyte hemolysis test, optical density reading values of the supernatant (Figure 11) were analyzed. Percentage of hemolysis observed was less than 1% in all investigated dilutions and sizes of nanoparticles. The percentage of hemolysis (mean ± SE) for 53 nm AuNPs was 0.25 (±0.07), −0.25 (±0.02), −0.25 (±0.01) and −0.25 (±0.01); and for 2 nm AuNPs was −0.25 (±0.01), −0.24 (±0.01), −0.24 (±0.01) and −0.26 (±0.00); at 1:1, 1:2, 1:5 and 1:10 dilutions, respectively. A significant difference was observed between 53 nm 1:1 (127 µM) and all the other groups, all dilutions of 2 nm, and the other three 53 nm dilutions (Figure 11D).

## 4. Discussion

Studies on applications of nanoparticles in biology and medicine primarily analyze the effect of size, shape, concentration, and incubation time of nanoparticles, in addition to the effect on cell kinetics after binding or cellular uptake. In the present study, investigations were carried on cytotoxicity and incorporation of gold nanoparticles of two sizes and four different concentrations of spherical LA-AuNPs, which were not synthesized by combining chemical solutions. Laser ablation of a high-purity gold pellet using Nd:YAG laser in the presence of distilled water produced the LA-AuNPs of two sizes. LA-AuNPs with 2 nm and 53 nm, at 84 μM, 42 μM and 23 μM, did not induce significant cytotoxicity in ASCs. However, a significantly lower median cell viability rate of 84% was observed after incubation with the larger nanoparticles at the highest concentration of 127 μM.

The production of nanoparticles with a wide size range distribution in the same sample is observed in chemically synthesized [21,27,30] and laser ablated AuNPs [34]. Laser ablation usually leads to broader size range dispersions when compared to chemically based techniques [38], which might be considered as a limitation of the AuNPs used in the present study. However, PLAL has the advantage of using a gold pellet target in aqueous media, without the addition of any other chemical; therefore, PLAL can be considered a “green approach” for nanoparticle preparation, particularly for biomedical applications [37].

The effect of AuNPs sterilization [56] and incubation with different cell types has been verified and reported by various studies [10,21,22,23,24,26,28,29,30,31,34,49,50,57]. The demonstrated ASCs great potential for cell transplant in bone tissue engineering, with the recognized osteogenic effect by nanoparticles and nanostructured scaffold materials [58], have already being translated into clinical treatments of mandibular and cranial bone defect [59,60]. The isolation of adipose tissue-derived mesenchymal stem cells or adipose tissue stromal vascular fraction can provide a reliable and abundant source of stem cells with neovascularization and osteogenic potential [54,61,62], possibly with nonimmunogenic cells from an autologous origin, indicated to improve bone healing using tissue engineering techniques.

AuNPs cellular uptake has been dependent on time, particle size, shape, and concentration [30,63]. The influence of the LA-AuNPs incubation on ASCs behavior was evaluated with serial photographs for 24 h. The possible mechanical stimulation from the adsorption of AuNPs with varying size and concentration onto the cells, may have determined the cellular response. The uptake of both LA-AUNPs sizes, in concentrations from 127µM to 23 µM (0.025 mg·mL^−1^ to 0.0045 mg·mL^−1^), allowed tracking the most labelled cells, which were observed in inverted light microscopy after incubation with nanoparticle supplementation medium. The cellular uptake was not uniform on all the cells observed on the specific aeras, only a few cells per area presented a distinguishable accumulation of LA-AuNPs. The amount of internalized nanoparticles has been shown to differ in a cell population, being higher as the cell advances through the cell cycle phase [64], and is influenced by the cell culture media, if basal or induction medium [31].

Light microscopy was used to access cell morphology and ASCs labeling with LA-AuNPs during cell culture. The dispersed internalization of small dots in the cytoplasm observed by light microscopy are described as dispersed small clusters in this study. The cellular uptake of a large perinuclear cluster of 2 nm and 53 nm LA-AuNPs, localized in the cytosol adjacent to the nucleus, was easily noticed and has been described previously [26,30]. Extracellular matrix (ECM) clusters were also noticed with spherical AuNPs by other authors [63].

The 24-h period for AuNPs incubation time, used by other researchers [10,23,50], was selected based on the observation that the cellular uptake was significantly higher in the first two hours and gradually reached a plateau between 4 h and 7 h [25]. However, longer incubation periods may promote higher AuNPs internalization, with greater number and diameter of forming vacuoles [30]. The cell-dependent variations on AuNPs cellular uptake determines the need of protocols with exposure time and concentration to optimize labelling efficacy with minimal impact on cell function and viability [22]. Size and concentration are interconnected factors which presented a significant effect on cellular degree of internalization of the LA-AuNPs and response. The exposure of ASCs to LA-AuNPs of two different sizes did not substantially affect cell viability, only with large nanoparticles at the highest concentration tested in the present study. Authors observed that cytotoxicity was concentration-dependent [21], and resulted also from the higher amount of sodium citrate, but not from the AuNPs’ size [28].

Substances which interfere with optical density detection, as possibly observed with adsorption and uptake of 53 nm at 127 µM LA-AuNPs, in the MTT assay, may result in a misinterpreted increase in mitochondrial activity (Supplementary material). Investigations with more than one assay are recommended, because of the interference of specific NPs with some toxicity methods [28,33,65,66,67]. A third colorimetric or luminogenic cytotoxicity assay, and their correlation with cell counting, would facilitate the identification of possible NPs’ interference [68], or cell death [69]. The other assays used, Live/Dead assay and the erythrocyte hemolysis test, demonstrated that 53 nm 127 µM presented the highest cell death and erythrocyte hemolysis values, respectively.

The nanoparticle uptake variation between spherical 53 nm compared to 2 nm LA-AuNPs was not tested in the present study but verified by others with 50 nm transferrin-coated particle compared to a size range of AuNPs [25,65,70]. The possible endocytic processes involved in cellular uptake of particles depend on activation stimulus from cell membrane receptor binding, actin polymerization, cytoskeleton organization, macropinosome, phagosome, intracellular vesicle formation [19,30,46,65,70,71,72], and intracellular degradation [73]. Different uptake behavior depending on cell types has been reported between epithelial and endothelial cells, supporting possible variations on the nanoparticle interaction with different cell membranes [28]. Further studies may identify the pathways involved in mediating the intracellular uptake of LA-AuNPs, besides subcellular events, degradation, and exocytosis. The preservation of LA-AuNPs cell labelling after cell detachment technique with trypsin was observed in the present study, which is fundamental for cell transplantation and in vivo tracking of those labelled cells. Authors have demonstrated that photoacoustic properties of AuNPs-labeled cells remained after freezing, storage, and thawing [23].

ASCs labeling with LA-AuNPs persisted for up to 2 weeks but was not quantified in this study. The possibility of nanoparticle exocytosis, degradation, or separation by cell division during the in vitro period should also be investigated, because a possible decrease in nanoparticle loading over time reduces the cell tracking strategy success. AuNPs have been detected by photoacoustic imaging from the ASCs-cultured gels for more than 2 weeks [50], and from AuNPs labelled ASCs after freezing, storage for up to 2 months, and thawing [23]. The recovery period of ASCs in AuNPs-free medium increased cell proliferation rates and recuperated adipogenic differentiation capacity but presented a reduction of AuNPs content in the progeny [31]. Higher exocytosis was observed in smaller AuNPs compared to larger particles [65]. The nanoparticle splitting between two cells in the process of mitosis has also been examined [30,64].

Cytoskeleton actin fibers distribution and integrity, which control cell shape, migration, and division, guarantee the investigation of cell morphology alteration with reduction of cell area resulting from the incubation, uptake of AuNPs, and cell recovery in AuNPs-free media. The recovery period of 1 and 2 weeks after LA-AuNPs incubation allowed the visual observation that cell shape and density was similar to control, without noticeable damage to actin filaments. It has been observed that HeLa cell culture monolayers had normal growth, without cell proliferation inhibition, change in cell morphology, or damage to f-actin microfilaments, caused by AuNPs incubation [26]. Minimal effect on cell viability was reported after AuNPs labelling because cell proliferation was not affected [22]. However, endothelial cell viability and the proliferation decreased after high concentrations of AuNPs exposure [28].

The observed fine dispersion of adsorbed particles on the intercellular spaces of the well surface, also observed by others [27], may have prevented cell motility and adhesion on the LA-AuNPs covered surface, decreasing the cell substrate area. A study with Human dermal fibroblasts (HDFs) has described that the presence of ‘loose’ particles interfered with actin fibers linkage with the ECM via integrins [30]. Cell exposure to AuNPs reduced HDFs’ cell area, the expression of collagen and fibronectin [30], and the migration of HDFs and ASCs [30,31].

Nanoparticle uptake effect on cell function and differentiation has been investigated [23,24,29,31,47,63] because the labelled and stored cells of viability and multipotency are essential for cell therapy applications. Nanoparticle loading has not been cytotoxic to mesenchymal stem cells and has not affected cell viability, and differentiation into adipogenic and osteogenic lineages [24], even after gold nanosphere-labeled ASCs have been frozen, stored for one week, one month, or two months, and then thawed [23]. However, others have reported that AuNPs inhibited adipogenic differentiation and stimulated osteogenic differentiation [47]. AuNPs increased cell population doubling times of HDFs and ASCs, decreased cell motility, cell-mediated collagen contraction, and reduced ASCs adipogenesis [30,31]. ASCs previously exposed to AuNPs for 2 weeks underwent cell function recovery and adipogenesis comparable to control cultures, after removal of exogenous AuNPs source [31]. The transplantation of ASCs on decellularized amniotic membranes demonstrated their potential for in vivo regeneration of bone defects [52], and a cell nanotracer which does not interfere with osteogenic differentiation may facilitate the tracking of these transplanted cells and the identification of possible cell contribution in the healing process. Approaches that utilize cells as components of a therapy present the inherent challenges of ex vivo graft preparation and in vivo investigation of cell influence and function. Initially, cell tracking after seeding on membranes and scaffolds is essential for evaluating cell response to potential three-dimensional cell carriers before their in vivo application. The investigation of ASCs after AuNPs uptake on a tridimensional polyethylene glycol modified fibrin gel system has allowed a long-term cell tracking with ultrasound and photoacoustic imaging, and observed no negative effect on cell morphology, viability, proliferation, network formation, and on expression and secretion of angiogenesis-related proteins and pericyte markers; maintaining the ASCs paracrine effect [50]. Gold nanoparticles have been used in tracking AuNPs labeled cells transplanted into animal models, using ultrasound and photoacoustic imaging [10,13,51], also for real time guided cell delivery [13], and Micro-Computed Tomography [11,12,49].

Further studies are recommended with LA-AuNPs, including cellular uptake assays [63], quantification of internalized gold nanoparticles [28], subcellular/intracellular distribution [13,22,31], viability after being labelled, frozen, stored, and then thawed [23], etc. The in vitro impact of LA-AuNPs on ASCs osteogenic differentiation, response to culture on collagenous matrix, and the in vivo cell tracking of transplanted ASCs labeled with La-AuNPs on amniotic membrane in bone defects, are some of the highly recommended investigations.

## 5. Conclusions

AuNPs prepared by laser ablation in aqueous solution, without the combination of chemical solutions, and functionalized using albumin, were biocompatible and safe for ASCs tracking. This preliminary in vitro study established safety viability cell parameters under which spherical AuNPs with two different sizes, but with a wide size range distribution, can be used for stem cell tracking in tissue engineering applications. Cellular uptake was more pronounced through exposure to larger AuNPs concentration and cell labelling persisted for up to two weeks.

## Data Availability

All data generated or analyzed during this study are included in this submitted article and its supplementary information file.

## Supplementary Materials

The following supporting information can be downloaded at https://www.mdpi.com/article/10.3390/ma16031034/s1 with Text of MTT Proceedings and Figure S1. Effect of Laser ablated Albumin functionalized AuNPs (LA-AuNPs) on Adipose-derived stromal cells (ASCs) metabolic activity, assessed by MTT, presented as survival percentage.
