# Supplementary material for: Laser Ablated Albumin Functionalized Spherical Gold Nanoparticles Indicated for Stem Cell Tracking
Source: Materials (Basel). 2023 Jan 24;16(3):1034. doi: 10.3390/ma16031034 (PMC9919444; doi:10.3390/ma16031034)
Supplement: Supplementary file 1 [file materials-16-01034-s001.zip › materials-2077055-supplementary.pdf]

Supplementary material

Laser Ablated Albumin Functionalized Spherical Gold Nanoparticles Indicated for Stem Cell Tracking

Dilcele Silva Moreira Dziedzic [1], Bassam Felipe Mogharbel [1], Ana Carolina Irioda [1], Priscila Elias Ferreira Stricker [1], Thiago Demetrius Woiski [1], Thiago Neves Machado [2], Arandi Ginane Bezerra Jr [2] and Katherine Athayde Teixeira de Carvalho [1,*]

MTT Proceedings

2. Materials and Methods

Metabolic activity assay

The metabolic activity of the Adipose-derived stromal cells (ASCs) exposed to Laser ablated and Albumin functionalized spherical gold nanoparticles (LA-AuNPs) was assessed based on mitochondrial cell function (dehydrogenases) by the reduction of the yellowish tetrazolium salt (MTT, [3- (4,5-dimethylthiazol-2yl) -2,5- diphenyl tetrazolium]), into formazan blue crystals (E, Z-1- (4,5-dimethylthiazol-2-yl) -1,3-diphenylformazan) insoluble in aqueous solution. ASCs were incubated for 24 hours with LA-AuNPs with average sizes of 2 nm and 53 nm in diameter, in four concentrations, 127μM, 84μM, 42μM, and 23μM. 96-well plates were prepared for investigation of ASCs viability with MTT after incubation with nanoparticles, including wells without cells and without LA-AuNPs as blanks, and with cells without LA-AuNPs as controls. After 24 hours incubation with LA-AuNPs, the medium was removed, the nanoparticles that were not incorporated into the cells or adhered to the well were removed with three washes of the wells with PBS, before new culture medium was added, without FBS and with MTT dilution. Briefly, culture medium solution with MTT (0.5 mg/ml) was placed in the wells and incubated for 3 hours. The medium was removed and replaced by DMSO, kept under gentle agitation for 5 minutes before reading the optical density in an ELISA reader with an absorbance value of 595nm. The absorbance value was calculated by subtracting the mean values from control/blank. The data from the MTT assay were analysed using Shapiro Wilk, Kruskal-Wallis, and multiple comparisons tests (STATISTICA software, StatSoft, Dell). Differences were considered significant when $p < 0.05$.

3. Results

After incubation with 2 nm and 53 nm LA-AuNPs for 24 hours, the metabolic activity of cells was compared to untreated cells by optical density percentage in MTT assays. The MTT method presented contradictory results in the observations of LA-AuNPs in cell culture, due to the increase in the optical density in the group of LA-AuNPs 53 nm at 1:1 dilution. As shown by the MTT assays, cell metabolic activity supposedly did not decrease with exposure to LA-AuNPs. Kruskal-Wallis's test for MTT assays was significant ($p = .0020$) and multiple comparisons observed differences between the percentage value of 53 nm LA-AuNPs at 1:1 dilution, versus 2 nm at 1:5, 2 nm at 1:10, 53 nm at 1:5, 53 nm at 1:10 and control (Figure S1). The possible interference of particles on the well surface, adhered to cell surface or due to the cellular uptake cannot be ruled out to be directly related to the inconsistent increase of optical density in MTT, higher than 100%.

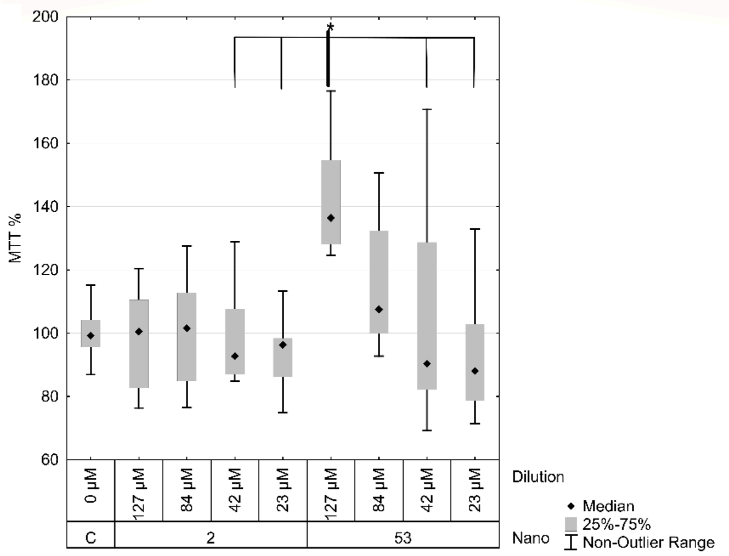

Figure S1. Effect of Laser ablated Albumin functionalized AuNPs (LA-AuNPs) on Adi-pose-derived stromal cells (ASCs) metabolic activity, assessed by MTT, presented as survival percentage. Cells were exposed for 24 hours to four different concentrations of nanoparticles with two different particle sizes, 2 nm, and 53 nm, in four dilutions: Control (C), 1:1 (127 µM), 1:2 (84 µM), 1:5 (42 µM) and 1:10 (23 µM). Data analyzed by Kruskal-Wallis's test and represented as median ± 25%-75%.

4. Discussion

The most common cell viability assays used are the metabolic activity MTT assay (Fraga et al., 2013; Ricles et al., 2011), Live Dead assays (Ricles et al., 2011; Ricles et al., 2014), LDH cell membrane integrity, immunohistochemistry biomarkers for apoptosis, and comet assay for genotoxicity (Bahadar et al., 2016; Fraga et al., 2013). Cytotoxicity of AuNPs has been observed to be shape-dependent, nanospheres were the safest, compared to nanorods and nanostars, the latter being the most cytotoxic as detected by MTT and Neutral red uptake viability assays (Steckiewicz et al., 2019). This study with LA-AuNPs corroborated with others, where AuNPs have not induced significant cytotoxicity when evaluated with MTT reduction (Fraga et al., 2013; Ricles et al., 2011). The large nanoparticles at the highest concentration tested (53 nm at 127 µM) contributed to the contradictory MTT ultraviolet-visible spectrophotometry readings, significantly superior to the control sample (above 100%). Substances which interfere with optical density detection, as possibly observed with adsorption and uptake of 53 nm at 127 µM LA-AuNPs, may result in a misinterpreted increase of mitochondrial activity.

Investigations with more than one assay are recommended, because of the interference of specific NPs with some toxicity methods (Avila-Alejo et al., 2017; Bahadar et al., 2016; Freese et al., 2012; Monteiro-Riviere et al., 2009). A colorimetric or luminogenic cytotoxicity assay, and their correlation with cell counting, would facilitate the identification of possible NPs' interference (Braun et al., 2018).The other two assays used in the present study, Live/Dead assay and the erythrocyte hemolysis test, demonstrated that 53 nm 127 µM presented the highest cell death and erythrocyte hemolysis values, respectively.

References

Avila-Alejo, J.O., Gonzalez-Palomo, A.K., Plascencia-Villa, G., Jose-Yacaman, M., Navarro-Contreras, H.R., and Perez-Maldonado, I.N. (2017). Low cytotoxicity of anisotropic gold nanoparticles coated with lysine on peripheral blood mononuclear cells "in vitro". Environ Toxicol Pharmacol *56*, 210-218.

Bahadar, H., Maqbool, F., Niaz, K., and Abdollahi, M. (2016). Toxicity of Nanoparticles and an Overview of Current Experimental Models. Iran Biomed J *20*, 1-11.

Braun, K., Sturzel, C.M., Biskupek, J., Kaiser, U., Kirchhoff, F., and Linden, M. (2018). Comparison of different cytotoxicity assays for in vitro evaluation of mesoporous silica nanoparticles. Toxicology in vitro : an international journal published in association with BIBRA *52*, 214-221.

Fraga, S., Faria, H., Soares, M.E., Duarte, J.A., Soares, L., Pereira, E., Costa-Pereira, C., Teixeira, J.P., de Lourdes Bastos, M., and Carmo, H. (2013). Influence of the surface coating on the cytotoxicity, genotoxicity and uptake of gold nanoparticles in human HepG2 cells. J Appl Toxicol *33*, 1111-1119.

Freese, C., Uboldi, C., Gibson, M.I., Unger, R.E., Weksler, B.B., Romero, I.A., Couraud, P.O., and Kirkpatrick, C.J. (2012). Uptake and cytotoxicity of citrate-coated gold nanospheres: Comparative studies on human endothelial and epithelial cells. Part Fibre Toxicol *9*, 23.

Monteiro-Riviere, N.A., Inman, A.O., and Zhang, L.W. (2009). Limitations and relative utility of screening assays to assess engineered nanoparticle toxicity in a human cell line. Toxicol Appl Pharmacol *234*, 222-235.

Ricles, L.M., Nam, S.Y., Sokolov, K., Emelianov, S.Y., and Suggs, L.J. (2011). Function of mesenchymal stem cells following loading of gold nanotracers. Int J Nanomedicine *6*, 407-416.

Ricles, L.M., Nam, S.Y., Trevino, E.A., Emelianov, S.Y., and Suggs, L.J. (2014). A Dual Gold Nanoparticle System for Mesenchymal Stem Cell Tracking. Journal of materials chemistry B, Materials for biology and medicine *2*, 8220-8230.

Steckiewicz, K.P., Barcinska, E., Malankowska, A., Zauszkiewicz-Pawlak, A., Nowaczyk, G., Zaleska-Medynska, A., and Inkielewicz-Stepniak, I. (2019). Impact of gold nanoparticles shape on their cytotoxicity against human osteoblast and osteosarcoma in in vitro model. Evaluation of the safety of use and anti-cancer potential. Journal of materials science Materials in medicine *30*, 22.
